# Impact of Gender and Race on Gastrointestinal Diseases in Patients With Parkinson's Disease: A Nationwide Analysis

**DOI:** 10.1002/jgh3.70302

**Published:** 2026-01-22

**Authors:** Mohamed H. Eldesouki, Ahmed Yasser Shaban, Mohamed Mahmoud Marey, Eslam Mohammed Rabea, Abdulrhman Helal, Mohamed Seisa, Omar Abdelhalim, Hazem Abosheaishaa, Mohamed Khalaf

**Affiliations:** ^1^ Department of Internal Medicine New York Medical College, Saint Michael's Medical Center Newark New Jersey USA; ^2^ Department of Internal Medicine, Faculty of Medicine Kafrelsheikh University Kafrelsheikh Egypt; ^3^ Department of Internal Medicine, Faculty of Medicine Alexandria University Hospital Alexandria Egypt; ^4^ Faculty of Medicine Benha University Benha Egypt; ^5^ Department of Internal Medicine UNC Health Blue Ridge Morganton North Carolina USA; ^6^ Department of Internal Medicine Icahn School of Medicine at Mount Sinai Queens Hospital Center New York New York USA; ^7^ Department of Gastroenterology, Hepatology & Nutrition ECU Health Medical Center/Brody School of Medicine Greenville North Carolina USA

**Keywords:** disparities, gastrointestinal diseases, gender, parkinson's disease, races

## Abstract

**Background:**

Gastrointestinal (GI) diseases are common non‐motor features of Parkinson's disease (PD), significantly impacting quality of life. The impact of racial and gender disparities in this population remains underexplored. This study examines the association of race and gender with GI diseases in hospitalized patients with PD.

**Methods:**

A retrospective study using the National Inpatient Sample was conducted, including 124,345 hospitalized patients with PD. Multivariable logistic regression was used to assess associations between GI diseases and race/ethnicity (White, Black, Hispanic) and sex, adjusting for confounders.

**Results:**

Overall prevalence of GI diseases in PD was 48.49%. Compared with White males, Black males had higher odds of dysphagia (aOR 1.27, 95% CI 1.04–1.56), and both Black (aOR 2.19, 95% CI 1.48–3.36) and Hispanic patients (aOR 1.7, 95% CI 1.05–2.77) had significantly higher odds of gastrostomy tube placement. White females had significantly higher odds of gastroesophageal reflux (GERD) (aOR 1.35, 95% CI 1.25–1.46), constipation (aOR 1.34, 95% CI 1.23–1.59), gastroparesis (GP) (aOR 3.44, 95% CI 2.23–5.31), and IBS (aOR 3.1, 95% CI 2.2–4.2), but lower odds of dysphagia (aOR 0.82, 95% CI 0.74–0.91), compared with White males. Hispanic females (aOR 2.64, 95% CI 1.52–4.56, *p* < 0.01) and Asian males (aOR 2.82, 95% CI 1.47– 5.41, *p* < 0.01) demonstrated significantly higher odds of *H. pylori* compared with white males.

**Conclusion:**

Significant racial and gender disparities were observed in GI diseases among hospitalized patients with PD. White females showed higher odds of GERD, constipation, GP, and IBS, while Black and Hispanic patients were more likely to require gastrostomy tube placement. *Helicobacter pylori* infection was higher in Hispanic females and Asian males when comapred to White males. These differences highlight the need for tailored, equitable care strategies and further research to better understand and address disparities in PD‐related GI outcomes.

## 1 Introduction

Parkinson's disease (PD) is a progressive neurodegenerative disorder that primarily affects the central nervous system; it is characterized by tremors, rigidity, postural instability, and bradykinesia [1]. These motor symptoms are linked to α‐synuclein (αSyn)‐containing inclusion bodies (Lewy pathology; LP) and the progressive loss of dopaminergic neurons in the substantia nigra, leading to dopamine depletion in the striatum [1]. However, PD also manifests non‐motor symptoms, including GI dysfunction, which significantly impact disease progression and quality of life [2]. PD is classified into two forms: familial and sporadic. The familial form results from genetic mutations, including those in the αSyn gene [3, 4, 5, 6, 7, 8, 9], locus duplication [10, 11], or triplication [11, 12]. Conversely, the causes of sporadic PD remain unknown, though research suggests genetic and environmental factors [13, 14, 15, 16]. In the United States, the incidence and prevalence of PD differ among racial groups, suggesting a genetic influence [17, 18, 19, 20].

Braak and colleagues hypothesized that an unknown gut pathogen (virus or bacterium) could initiate sporadic PD development [21]. They suggested that misfolding and aggregation of αSyn occur in two locations: the nasal cavity neurons and the gut neurons [22, 23, 24, 25, 26]. Misfolded αSyn accumulation in the enteric nervous system (ENS) leads to GI symptoms such as dysphagia, nausea, constipation, and defecatory difficulty, often observed years before the onset of motor symptoms [27]. This pathology may spread to the brain via the vagus nerve [28]. Once αSyn reaches the brain, it spreads to various regions, causing the characteristic motor symptoms of PD [29].

GI diseases in PD vary in prevalence and nature across genders, races, and ethnicities. Constipation, delayed gastric emptying, dysphagia, and small intestinal bacterial overgrowth (SIBO) are among the most reported symptoms. However, associations between race, ethnicity, and GI symptom prevalence remain underexplored. Existing literature highlights gender‐based differences, with women reporting higher rates of nausea, bloating, and non‐motor symptoms, while men more commonly experience constipation [30, 32]. Racial disparities have also been noted, with African American and Hispanic patients with PD experiencing higher rates of comorbidities, including GI disorders, compared to their Caucasian counterparts. Nonetheless, most studies primarily focus on Caucasian populations, leaving underrepresented minorities with delayed diagnoses and reduced access to care [33]. This underrepresentation underscores a significant gap that must be addressed to fully understand disease heterogeneity.

This study's goals are to fill the existing gaps in the literature by using a nationwide database to examine the relationship between gender, race, and ethnicity, and the prevalence of GI diseases in patients with PD, thereby helping to make a more inclusive and personalized approach to the management of GI diseases in this population.

## 2 Methods

### 2.1 Study Design and Data Source

We conducted this retrospective study using the National Inpatient Sample (NIS) database from 2016 to 2021. As a component of the Agency for Healthcare Research and Quality and the Healthcare Cost and Utilization Project, NIS is the biggest longitudinal hospital care database in the US which comprises a stratified sample of discharges from more than 1000 general, short‐term, non‐federal, and specialty hospitals nationwide, accounting for about 20% of all hospitalizations each year [34, 35]. NIS delivers comprehensive patient‐level data, such as demographics, hospital characteristics, service consumption, and overall hospital expenses for research purposes in accordance with the International Classification of Diseases, Tenth Revision, Clinical Modification (ICD‐10‐CM) [35]. Our study follows HCUP Data Use Agreement guidelines [36]. Upon request, the corresponding author will provide the data supporting this study's findings. Institutional review board approval isn't required as the NIS database is public.

### 2.2 Study Population and Selection Criteria

Adult patients (≥ 35 years old) with PD who were admitted to hospitals were included. Based on self‐reported race and sex as documented in the NIS, the study sample was stratified by sex and ethnicity into ten subgroups: Black males, Black females, Hispanic males, Hispanic females, White males, White females, Native American males, Native American females, Asian males, and Asian females. Patients were categorized as having missing data if their sex was not disclosed, was unknown, or was non‐binary. Patients with missing data on race, sex, or hospital‐related data were excluded from our study. Our findings may not apply to patients excluded from the study, as their characteristics may differ from those of the included patients. All codes utilized in our study are listed in Table S1.

### 2.3 Baseline Characteristics and Outcomes

Demographic data included age, sex, and race/ethnicity. Our outcomes were GI disorders that included dysphagia, diarrhea, constipation, outlet dysfunction‐related constipation, gastroesophageal reflux disease (GERD), functional dyspepsia (FD), irritable bowel syndrome (IBS), incontinence, *Helicobacter pylori* ( *H. pylori* ), inflammatory bowel disease (IBD), gastroparesis (GP), intestinal obstruction (IO), and the need for placement of percutaneous endoscopic gastrostomy (PEG) tube. Most of these study outcomes and their codes were utilized in previous database studies for PD and GI disease [37].

### 2.4 Statistical Analysis

All data obtained were weighted following HCUP guidelines [38] and then analyzed using Stata Software version 17.0 (College Station, TX) [39]. Demographic data and the prevalence of GI diseases in PD patients across age groups were reported using descriptive statistics, including frequencies and percentages. The prevalence of GI disorders, complications, and procedures was reported for each subgroup and then compared using the Chi‐square test. Multivariable logistic regression models were done to assess the relationship between race/ethnicity, sex, and study outcomes adjusting for potential confounding by various comorbidities that included hypertension (HTN), diabetes mellitus (DM), liver diseases, coronary artery disease (CAD), cancer, dialysis dependency, alcohol use, obstructive sleep apnea (OSA), pulmonary diseases, dyslipidemia, stroke, smoking, and comorbidities captured by the Charlson Comorbidity Index (CCI). Adjusted odds ratios (aORs) with 95% confidence intervals (CIs) and *p*‐values were calculated to estimate the strength of associations within subgroups and assess statistical significance (*p* < 0.05). These results were visualized using forest plots to compare aORs and their CIs for each subgroup.

## 3 Results

### 3.1 Patient Baseline Characteristics

From 2016 to 2021, a total of 124, 345 patients with PD were identified, of whom 54.67% were male and 42.33% were female. The overall prevalence of GI diseases among patients with PD was 48.49%. Within this subgroup, 33.7% (20,324) were males. White race constituted the largest group at 80.31% (97,080); 52.31% (White males) and 25.75% (White females), followed by Black or African Americans (AA) and Hispanics at 7.12% (8610) and 6.97% (8430), respectively Asians were 2.6% (3385) while Native American were 0.31% (380). Most of the included patients in our study population aged between 66–75 years old (34.29%) and 76–85 years old (35.98%) (Tables S2 and S3). The comorbidities of the included population included HTN (47.09%) and DM (24.30%). Differences in socioeconomic status were also evident, with a higher proportion of Black and Hispanic patients belonging to the lowest income quartile compared with White patients. Medicaid was the primary payer for 12.09% of Black and 16.04% of Hispanic patients compared with 3.08% of White patients (Table 1). The prevalence of GI diseases was divided according to age groups. It was highly prevalent in the 66–75 years old group (36.23%) and 76–85 years old group (35.64%) while lower in the 35–45 years old group (0.65%), 46–55 years old group (3.98%), 56–65 years old group (13.2%), and ≥ 86 years old group (11.81%) as illustrated in Figure S1.

**TABLE 1:** Baseline characteristics for the patients included in the study.

Patient characteristics	White Males = 65,050	White Females = 32,020	Black Males = 5,200	Black Females = 3,405	Hispanic Males = 5,535	Hispanic Females = 2,890	Asian Male = 1,975	Asian Female = 1,210	Native American Male = 259	Native American Female = 120	*p*
Age											
35–65	11 070 (17.03)	4925 (15.4)	1040 (20.04)	690 (20.29)	1665 (30.08)	635 (22.09)	475 (24.05)	235 (19.42)	80 (30.89)	30 (25.00)	< 0.01
65–85	46 460 (71.49)	22 685 (70.91)	3650 (70.33)	2280 (67.06)	3460 (62.62)	1885 (65.57)	1265 (64.05)	860 (71.07)	165 (63.71)	85 (70.83)	< 0.01
> 85	7455 (11.47)	4380 (13.69)	500 (9.63)	430 (12.65)	500 (9.05)	355 (12.35)	235 (11.90)	110 (9.09)	15 (5.79)	5 (4.17)	< 0.01
Comorbidities											
Hypertension	29 530 (45.4)	15 400 (48.09)	2665 (51.25)	2000 (58.74)	2705 (48.87)	1435 (49.65)	869 (44)	573 (47.32)	132 (50.95)	63 (52.56)	< 0.01
Diabetes Mellitus	15 525 (23.87)	6200 (19.36)	1930 (37.12)	1275 (37.44)	1855 (33.51)	945 (32.7)	553 (28)	303 (25.02)	91 (35.23)	40 (33.11)	< 0.01
Alcohol use	1105 (1.7)	240 (0.75)	115 (2.21)	10 (0.29)	110 (1.99)	5 (0.17)	24 (1.2)	6 (0.51)	5 (2.05)	1 (1.0)	< 0.01
Liver disease	755 (1.16)	420 (1.31)	135 (2.6)	30 (0.88)	110 (1.99)	45 (1.56)	30 (1.5)	17 (1.41)	6 (2.21)	2 (1.7)	< 0.01
Obesity	5015 (7.71)	2660 (8.31)	235 (4.52)	295 (8.66)	300 (5.42)	240 (8.3)	119 (6.05)	85 (7.05)	25 (9.56)	10 (8.11)	< 0.01
Dialysis	265 (0.41)	115 (0.36)	100 (1.92)	50 (1.47)	45 (0.81)	30 (1.04)	14 (0.7)	6 (0.51)	3 (1.12)	2 (1.3)	< 0.01
Obstructive sleep apnea	6410 (9.85)	1495 (4.67)	250 (4.81)	105 (3.08)	260 (4.7)	65 (2.25)	104 (5.26)	37 (3.02)	10 (4.03)	4 (3.52)	< 0.01
Insurance (%)											
Medicare	54 213 (83.34)	27 864 (87.02)	4163 (80.06)	2815 (82.67)	4066 (73.46)	2284 (79.03)	1604 (81.23)	1018 (84.11)	213 (82.05)	102 (85.02)	< 0.01
Medicaid	989 (1.52)	500 (1.56)	331 (6.36)	195 (5.73)	456 (8.24)	225 (7.8)	121 (6.15)	61 (5.02)	18 (7.01)	7 (6.21)	< 0.01
Private	7780 (11.96)	3522 (11.00)	521 (10.02)	290 (8.52)	727 (13.13)	286 (9.88)	222 (11.22)	113 (9.36)	27 (10.31)	10 (8.09)	< 0.01
Non‐insured	338 (0.52)	154 (0.48)	70 (1.35)	45 (1.32)	110 (1.99)	35 (1.21)	46 (2.32)	10 (0.81)	4 (1.52)	1 (1.21)	< 0.01
Median Annual Income by Zip Code, (%)											
1st Quartile	11 969 (18.4)	6436 (20.1)	2233 (42.94)	1596 (46.88)	1932 (34.9)	1093 (37.83)	597 (30.21)	387 (32.01)	104 (39.98)	51 (42.42)	< 0.01
2nd Quartile	15 885 (24.42)	8091 (25.27)	1104 (21.23)	763 (22.4)	1447 (26.15)	633 (21.89)	478 (24.21)	318 (26.32)	65 (25.14)	28 (23.22)	< 0.01
3rd Quartile	18 162 (27.9)	8684 (27.12)	1012 (19.47)	586 (17.21)	1111 (20.07)	648 (22.42)	461 (23.35)	291 (24.02)	52 (20.2)	27 (22.5)	< 0.01
4th Quartile	19 040 (29.27)	8809 (27.51)	851 (16.36)	460 (13.5)	1045 (18.88)	516 (17.86)	457 (23.12)	220 (18.21)	39 (15.15)	16 (13.6)	< 0.01
Hospital Bed size, (%)											
Small	12 834 (19.73)	6490 (20.27)	1120 (21.54)	710 (20.85)	915 (16.53)	475 (16.44)	380 (19.23)	222 (18.33)	55 (21.26)	24 (19.94)	< 0.01
Medium	16 503 (25.37)	8549 (26.7)	1405 (27.02)	1001 (29.396)	1545 (27.91)	800 (27.68)	564 (28.56)	327 (27.06)	65 (25.11)	33 (27.15)	< 0.01
Large	35 712 (54.9)	16 980 (53.03)	2675 (51.44)	1675 (49.19)	3075 (55.56)	1615 (55.88)	1049 (53.12)	667 (55.14)	140 (54.23)	64 (52.98)	< 0.01
Hospital Teaching Status, (%)											
Non‐teaching	11 247 (17.29)	6295 (19.66)	985 (18.94)	514 (15.09)	1140 (20.6)	555 (19.2)	361 (18.28)	198 (16.38)	50 (19.43)	25 (20.86)	< 0.01
Teaching	48 235 (74.15)	22 753 (71.06)	4215 (81.06)	2755 (80.91)	4395 (79.4)	2335 (80.8)	1564 (79.21)	994 (82.13)	210 (81)	96 (80.21)	< 0.01
Charlson Comorbidity Index, (%)											
0	19 287 (29.65)	11 191 (34.95)	1012 (19.47)	759 (22.3)	1750 (31.61)	947 (32.77)	556 (28.15)	366 (30.25)	66 (25.36)	33 (27.46)	< 0.01
1	19 021 (29.24)	9971 (31.14)	1342 (25.81)	980 (28.78)	1625 (29.36)	812 (28.09)	540 (27.36)	345 (28.52)	69 (26.53)	30 (25.28)	< 0.01
2	11 416 (17.55)	5341 (16.68)	1084 (20.85)	625 (18.35)	897 (16.2)	516 (17.87)	381 (19.28)	216 (17.89)	54 (20.68)	23 (19.02)	< 0.01
≥ 3	15 326 (23.56)	5517 (17.23)	1761 (33.87)	1041 (30.57)	1264 (22.83)	615 (21.27)	517 (26.18)	294 (24.26)	76 (29.42)	35 (29.39)	< 0.01

### 3.2 Gastrointestinal Diseases in Parkinson's Patients

In our study, the overall prevalence of GERD among those with PD was 21.66%. The prevelance was highest in White males compared to the other groups (52.04% vs. 30.30% (White females); 2.93% (Black males); 2.40% (Black females); 3.14% (Hispanic males); and 2.27% (Hispanic females)). Multivariable logistic regression showed significantly higher odds of GERD in White female patients compared with White males (aOR 1.35, 95% CI 1.25–1.46, *p* < 0.01). However, GERD was significantly lower in Black males (aOR 0.65, 95% CI 0.54–0.79, *p* < 0.01) and Hispanic males (aOR 0.72, 95% CI 0.59–0.88, *p* < 0.01) (Table 2, Table S4).

**TABLE 2:** Multivariable logistic regression models assessing associations between race/ethnicity, sex and gastrointestinal diseases among patients with Parkinson's disease.

GI disease	Race/ethnicity and sex	aOR^b^	95% CI	*p*
GERD	White Female	1.35	(1.25–1.46)	**< 0.01**
	Black Males	0.65	(0.54–0.79)	**< 0.01**
	Black Female	0.84	(0.67–1.05)	0.13
	Hispanic Male	0.72	(0.59–0.88)	**< 0.01**
	Hispanic Female	1.04	(0.82–1.33)	0.72
	Asian Male	0.72	(0.52–0.99)	0.05
	Asian Female	0.71	(0.47–1.07)	0.103
	Native Male	0.83	0.34–2.03	0.7
	Native Female	1.32	0.50–3.50	0.32
Dysphagia	White Female	0.82	(0.74–0.91)	**< 0.01**
	Black Males	1.27	(1.04–1.56)	**0.02**
	Black Female	0.93	(0.72–1.21)	0.58
	Hispanic Male	1.29	(1.05–1.59)	**0.01**
	Hispanic Female	0.92	(0.69–1.24)	0.59
	Asian Male	1.07	0.77–1.49	0.67
	Asian Female	1.26	0.86–1.86	0.22
	Native Male	1.2	0.41–3.6	0.73
	Native Female	0.75	0.18–3.1	0.68
Gastroparesis^a^	White Female	3.44	(2.23–5.31)	**< 0.01**
	Black Males	1.17	(0.41–3.37)	0.77
	Black Female	2.18	(0.86–3.51)	0.1
	Hispanic Male	0.57	(0.48–2.85)	0.34
	Hispanic Female	2.15	(0.65–3.10)	0.21
	Asian Male	1.238	0.16–3.42	0.83
IBS^a^	White Female	3.06	(2.2–4.2)	**< 0.01**
	Black Males	0.63	(0.19–2.01)	0.43
	Black Female	0.95	(0.29–3.1)	0.93
	Hispanic Male	0.43	(0.11–1.76)	0.24
	Hispanic Female	1.2	(0.38–3.9)	0.74
Constipation	White Female	1.34	(1.23–1.59)	**< 0.01**
	Black Males	0.49	(0.35–0.71)	**< 0.01**
	Black Female	1.04	(0.76–1.43)	0.81
	Hispanic Male	0.68	(0.50–0.93)	0.01
	Hispanic Female	1.34	0.94–1.90	0.11
	Asian Male	0.9320	0.64–1.36	0.72
	Asian Female	1.379	0.90–2.113	0.14
	Native Male	1.44	0.48–4.34	0.52
	**Native Female**	**3.08**	**1.17–8.1**	**0.02**
Constiptaion related outlet dysfunction^a^	White Female	1.42	(0.96–2.11)	0.08
	Black Males	0.89	(0.33–2.36)	0.82
	Black Female	2.70	0.83–3.67	0.10
	Hispanic Male	0.87	0.21–3.82	0.86
	Hispanic Female	1.63	0.41–3.53	0.49
	Asian Female	3.63	0.86–4.16	0.07
Diarrhea^a^	White Female	1.17	(0.88–1.57)	0.26
	Black Males	0.63	(0.29–1.4)	0.237
	Black Female	0.83	(0.37–1.85)	0.64
	Hispanic Male	0.57	(0.25–1.3)	0.18
	Hispanic Female	1.77	(0.91–3.44)	0.09
	Asian Male	1.08	0.38–3.11	0.87
	Asian Female	0.85	0.20–3.56	0.83
Fecal incontinence^a^	White Female	0.81	(0.56–1.16)	0.3
	Black Males	1.29	(0.68–2.44)	0.4
	Black Female	1.48	(0.71–3.1)	0.2
	Hispanic Male	1.04	(0.49–2.16)	0.9
	Hispanic Female	0.69	(0.21–2.2)	0.5
	Asian Male	1.628	0.64–4.15	0.30
	Asian Female	0.532	0.07–3.84	0.53
IBD^a^	White Female	1.20	0.81–1.79	0.36
	Black Female	1.41	0.52–3.86	0.49
	Hispanic Male	0.21	0.03–1.52	0.12
	Asian Male	3.87	0.48–4.91	0.36
Functional dyspepsia^a^	White Female	1.45	(0.67–2.54)	0.2
	Hispanic Male	1.61	(0.19–3.86)	0.6
Intestinal obstruction	White Female	0.62	(0.41–0.92)	**0.019**
	Black Males	1.64	(0.90–2.9)	0.108
	Black Female	0.97	(0.39–2.37)	0.94
	Hispanic Male	1.02	(0.49–2.14)	0.94
	Hispanic Female	0.69	(0.22–2.22)	0.54
	Asian Male	0.33	0.04–2.35	0.26
	Asian Female	1.6398	0.50–5.35	0.41
	Native Male	3.28	0.44–4.1	1.17
*H. pylori*	White Female	1.13	0.87–1.47	0.35
	Black Males	0.92	0.49–1.73	0.80
	Black Female	1.58	0.86–2.91	0.136
	Hispanic Male	1.37	0.78–2.41	0.26
	Hispanic Female	2.64	1.52–4.56	**0.001**
	Asian Male	2.82	1.47–5.41	**0.002**
	Asian Female	0.41	0.05–3.10	0.3
	Native Female	3.97	(0.59–4.4)	0.15
Patient who needed PEG	White Female	0.92	(0.70–1.22)	0.6
	Black Males	2.19	(1.48–3.36)	**< 0.01**
	Black Female	2.82	(1.78–4.56)	**< 0.01**
	Hispanic Male	1.7	(1.05–2.77)	**0.028**
	Hispanic Female	1.97	(1.13–3.55)	**0.017**
	Asian Male	1.17	0.51–2.67	0.70
	Asian Female	1.90	0.81–4.52	0.143

Abbreviations: aOR, adjusted odds ratio; CI, confidence interval; FD, functional dyspepsia; FI, fecal incontinence; GERD, gastroesophageal reflux disease; GI, gastrointestinal; *H. pylori* , *Helicobacter pylori* ; IBD, inflammatory bowel disease; IBS, irritable bowel syndrome; OD, outlet dysfunction; PEG, percutaneous endoscopic gastrostomy.

^a^ Missing subgroups were omitted from regression models because of sparse case counts for several outcomes, which precluded stable estimation.

^b^ Reference group was White males, a OR (adjusted odds ratio).

The overall prevalence of dysphagia among our population was 12.88%. The prevalence was highest in White males and White females 54.28% and 22.67%, respectively. After multivariable logistic regression, there were higher odds of dysphagia in Black males (aOR 1.27, 95% CI 1.04–1.56, *p* = 0.02), and Hispanic males (aOR 1.29, 95% CI 1.05–1.59, *p* = 0.01), while lower in White females (aOR 0.82, 95% CI 0.74–0.91, *p* < 0.01) compared with white males (Table 2, Table S4).

Constipation prevalence in PD patients was 7.39% with the highest prevalence in white males (54.54%) and white females (28.93%) compared with other groups. The aOR of constipation was higher in white females (aOR 1.34, 95% CI 1.23–1.59, *p* < 0.01) but lower in black males (aOR 0.49, 95% CI 0.35–0.71, *p* < 0.01), and Hispanic males (aOR 0.68, 95% CI 0.50–0.93, *p* < 0.01) when compared with white males (Table 2, Table S4). The prevalence of outlet dysfunction–related constipation was 1.2%, with no significant differences observed between groups. *Helicobacter pylori* infection and related gastritis were present in 4.15% of patients, and no statistically significant differences were detected across the study cohorts. The prevalence of *H. pylori* infection and related gastritis among PD patients was 2.12%. In adjusted analyses, Hispanic females (aOR 2.64, 95% CI 1.52–4.56, *p* < 0.01) and Asian males (aOR 2.82, 95% CI 1.47– 5.41, *p* < 0.01) demonstrated significantly higher odds of *H. pylori* compared with white males. GP and IBS had a low prevalence among our hospitalized population (0.46%) and (0.90%), respectively. Both had the highest prevalence in white females (50.43% GP, 53.13% IBS). Our analysis showed that GP and IBS are significantly higher in white females up to three times when compared to white males (aOR 3.44, 95% CI 2.23–5.31, *p* < 0.01) and (aOR 3.06, 95% CI 2.2–4.2, *p* < 0.01), respectively (Table 2, Table S4). Other GI diseases including diarrhea, fecal incontinence, IBD, and FD have a very low prevalence in PD patients. Multivariable logistic regression showed no statistically significant differences between the groups in any of these diseases (Table 2, Table S4). Intestinal obstruction prevalence was 0.81% among the included population. White males were more likely to have IO compared to the other groups (58.21% vs. 16.42% [white females]; 6.97% [black males]; 2.49% [black females]; 4.98% [Hispanic males]; and 1.99% [Hispanic females]). In the adjusted analysis, white females with PD had significantly lower odds of IO (aOR 0.62% CI 0.41–0.92, *p* = 0.019) compared to white males while no significant difference was reported between the other groups (Table 2, Table S3). The prevalence of patients who needed a percutaneous gastrostomy tube placement was 1.61% with the highest prevalence in white males (41.90%). After applying multivariable logistic regression, it showed that black females had the highest odds of PEG insertion compared with white males (aOR 2.82, 95% CI 1.78–4.56, *p* < 0.01). The aOR of inserting a gastrostomy tube was also higher in black males (aOR 2.19, 95% CI 1.48–3.36, *p* < 0.01), Hispanic males (aOR 1.7, 95% CI 1.05–2.77, *p* = 0.028), and Hispanic females (aOR 1.97, 95% CI 1.13–3.55, *p* = 0.017) compared with white males (Table 2, Table S4).

## 4 Discussion

Our study showed that white female patients with PD had higher odds of GERD, GP, IBS, and constipation, while having fewer odds of dysphagia compared with white males. Black and Hispanic patients with PD recorded a higher need for gastrostomy tube placement when compared with white males. Black and Hispanic males with PD had higher odds of dysphagia, and lower odds of GERD when compared with white males. No significant differences were noted in the odds of functional dyspepsia, diarrhea, fecal incontinence, or inflammatory bowel diseases. The odds of intestinal obstruction were significantly lower in white females compared with white males.

GI dysfunction is a common non‐motor symptom associated with PD [40]. This association is bidirectional and may represent either an integral aspect of the disease or an early prodromal phase preceding motor symptom onset. Pathological studies indicate that accumulation of aSyn aggregates in the ENS can occur up to 20 years prior to the onset of PD motor symptoms [41, 42]. Konings et al. found that long‐term dysphagia, GP, IBS without diarrhea and constipation might specifically predict the future occurrence of PD. The Enteric Nervous System represents the gateway for the bidirectional association between PD and the GI system [43]. Research suggested that gut dysbiosis may play a role in triggering the formation of aSyn aggregates in the ENS, potentially acting as the trigger for PD. Furthermore, GI diseases can emerge as one of the neurological consequences in the later stages of PD [44, 45]. The significance of studying GI dysfunction in PD lies in several aspects. It offers insight into early indicators of the disease and paves the way for early identifications and novel interventions, including dietary modifications and microbiome‐targeted therapies [45]. Non‐motor symptoms, such as GI dysfunction, have been reported to affect the quality of life in some cases more significantly than motor symptoms [46]. Furthermore, GI dysfunction can interfere with PD treatment by impacting the function and absorption of drugs like levodopa [47].

Due to the variability in clinical practices for managing PD and its related disorders, we aimed to explore ethnic, racial, and sex‐based disparities in GI diseases in PD patients in the United States. By recognizing these disparities, we can pinpoint the groups who need special attention and proactive screening. This can also improve early detection of PD by identifying groups with higher odds of GI diseases. In addition, we can highlight the presence of any possible health inequities by identifying disparities in complications. In this study, approximately 40% of PD patients admitted to hospitals between 2016 and 2021 exhibited GI diseases. This percentage likely underestimates the true prevalence, as it includes only hospitalized patients. Previous studies have reported prevalence rates as high as 80% [45, 48]. The variability in reported prevalence may be attributed to differences in the definitions and criteria used for GI diseases across studies.

Black and Hispanic males had fewer odds of GERD compared with white males. These findings align with reported racial disparities regarding GERD. Previous studies demonstrated that Black individuals appear to be at a lower risk for the development of GERD [49]. Regarding dysphagia, Black and Hispanic males had higher odds compared with white males. We also observed that white women had higher odds of IBS, constipation, and GP compared with white males. Interestingly, these disparities align with patterns reported in studies focusing on these GI disorders as standalone conditions, independent of PD [50, 52]. This identifies the female gender as a risk factor for GERD, GP, IBS, and constipation. Additionally, Black and Hispanic males with PD are more prone to dysphagia. IO was less likely to occur in females compared with males. The male predominance for these two complications was reported in previous studies [53, 54].

Previous literature has highlighted the racial disparities in PD in terms of different facets: knowledge about the disease, access to care, problems in diagnosis, physician referral, and the provided treatment [55, 57]. Luca et al. recently investigated variability in health‐related quality of life (HRQoL) in PD patients across different races and ethnicities. It's reported worse HRQoL among non‐White patients when compared with White patients. This may explain the observed higher rates of PEG tube placement in Black and Hispanic patients when compared to White males. Insertion of the PEG tube is often used in advanced stages of PD for long‐term enteral nutrition when patients experience significant difficulties with swallowing, malnutrition, or dehydration. However, survival after PEG was reported to be short and aspiration pneumonia was not prevented. Our study showed higher odds of PEG tube placement in Black and Hispanics when compared to white males.

The disparities reported in this study arise as a result of multiple interacting factors. Disparities in GI diseases like GERD, IBS, constipation, and GP may be yielded from inherent disparities due to genetic variations, patients' comorbidities, and health‐seeking behaviors that may rather affect the overall presentation and management of GI diseases [55]. Additionally, implicit bias, and disparities in clinical decision‐making may affect recognition and treatment of PD and its related GI diseases. For instance, some providers may have a bias toward instituting complex interventions like PEG tube placement in certain racial or ethnic groups. Furthermore, disparities in access to specialized care, differences in insurance coverage, and regional variations in healthcare infrastructure and practices may lead to these disparities [58].

Our study calls for individualized patient care programs to mitigate disparity in GI diseases. Identifying patients at higher risk based on demographic and clinical factors is essential for early detection and targeted management. Future research efforts are warranted to find the sources behind inequality and to ascertain their modifiability status. Addressing modifiable factors of inequities will require multi‐faceted strategies, such as raising patients' awareness about PD and its related non‐motor disorders, enhancing access to care through policy interventions, and increasing awareness about racial and sex‐based differences in disease patterns. By fostering equitable practices, we can improve the quality of care for vulnerable populations and ensure better health outcomes for all individuals with PD.

The strengths of our study include the use of a very large and nationally representative dataset, which allows robust statistical analyses and enhances the generalizability of findings beyond the study sample. The large sample size also provided sufficient power to detect real differences across subgroups, and the use of multivariable regression models helped control for several potential confounders, increasing our confidence in the findings. Importantly, the NIS dataset enabled us to capture several 100 Asian, Native American, Black, and Hispanic patients with Parkinson's disease—numbers that would be very difficult to achieve in a single‐center study. This provides valuable preliminary insights into gastrointestinal manifestations among minority populations.

However, several limitations should be acknowledged. As with any study utilizing administrative data, there is potential for coding errors, missing data, and misclassification of diagnoses, procedures, and outcomes, which may affect the accuracy of our results. The dataset's reliance on hospital‐based coding also limits the availability of detailed clinical information, such as the timing and progression of GI symptoms. Furthermore, because the NIS dataset is cross‐sectional and does not provide longitudinal information, we were unable to determine whether GI disorders occurred before or after the diagnosis of PD. This prevents us from establishing a temporal sequence or causality between PD and associated GI outcomes. Another limitation is the relatively small number of Asian and Native American patients and the smaller proportion of Black and Hispanic patients compared with White patients. Although these groups were included in descriptive analyses, subgroup sizes were often too small to support stable regression estimates, leading to omission in multivariable analyses, according to HCUP/NIS recommendations. As such, the findings may not be fully generalizable to these populations. Future studies should embrace a broader spectrum of racial and ethnic groups and ideally incorporate validated clinical assessments to strengthen accuracy.

## 5 Conclusion

Our study highlights significant racial, ethnic, and sex‐based disparities in the prevalence of GI diseases and complications among hospitalized patients with PD in the US. Compared with White males, White females had higher odds of GERD, gastroparesis, IBS, and constipation. Black and Hispanic patients with PD were more likely to undergo gastrostomy tube placement, while *H. pylori* infection was significantly more common in Hispanic females and Asian males. These patterns underscore the importance of developing tailored care approaches and conducting further research to better understand and address disparities in GI outcomes among diverse PD populations.

## Funding

The authors have nothing to report.

## Conflicts of Interest

The authors declare no conflicts of interest.

## Supporting information


**Data S1:** Supporting Information.

## Data Availability

You state data are available from the corresponding author upon request, but NIS is obtained through HCUP and governed by the HCUP DUA.
