# Supplementary material for: Impact of Gender and Race on Gastrointestinal Diseases in Patients With Parkinson's Disease: A Nationwide Analysis
Source: JGH Open. 2026 Jan 22;10(1):e70302. doi: 10.1002/jgh3.70302 (PMC12827495; doi:10.1002/jgh3.70302)
Supplement: Supplementary file 1 — Data S1: Supporting Information. [file JGH3-10-e70302-s001.docx]

# **Table S 1 : Codes utilized in our study**

- B9681 — Helicobacter pylori infection
- R197 — Diarrhea
- K21 — Gastroesophageal reflux disease (GERD)
- K30 — Functional dyspepsia
- K3184 — Gastroparesis
- K50 — Crohn’s disease (CD)
- K51 — Ulcerative colitis (UC)
- K56 — Intestinal obstruction
- K58 — Irritable bowel syndrome (IBS)
- K5902 — Outlet dysfunction constipation
- K59.0 — Constipation
- R13 — Dysphagia
- R14 — Abdominal bloating
- R15 — Fecal incontinence
- R197 — Diarrhea, unspecified
- Insertion of feeding gastrostomy tube - 0DH50UZ 0DH60UZ 0DH63UZ 0DH80UZ 0DH90UZ 0DH83UZ 0DH68UZ 0DH64UZ 0DH67UZ 0DH53UZ 0DH54UZ

Table .S2 Number and percentage and all individuals who were included in our study

| **Variable** | **N (%)** |
| --- | --- |
| **RACE** |  |
| White | 97080 (80.31) |
| African Americans | 8610 (7.119) |
| Hispanics | 8430 (6.970) |
| Asian or Pacific Islander | 3185 (2.635) |
| Native American | 380 (0.31) |
| Others | 3210 (2.65) |
| **Gender** |  |
| Female | 42,687 (42.33) |
| Male | 67,979 (54.67) |

Table .S3 Age groups of patients of Parkinson's disease who were admitted to the hospitals through 2016 to 2021

| Age group | N | Percentage |
| --- | --- | --- |
| <35 | 150 | 0.12 |
| 35 - 45 | 805 | 0.65 |
| 46 - 55 | 4410 | 3.55 |
| 56 - 65 | 16935 | 13.62 |
| 66- 75 | 42635 | 34.29 |
| 76-85 | 44740 | 35.98 |
| >= 86 | 14670 | 11.81 |

Table .S4 Unadjusted percentage of gastrointestinal diseases and complications based on their associations with race/ethnicity and sex.

| **Variables** | **Total** | **White Males = 65050** | | **White Females = 32020** | | **Black Males = 5200** | | **Black Females = 3405** | | **Hispanic Males = 5535** | | **Hispanic Females = 2890** | |
| --- | --- | --- | --- | --- | --- | --- | --- | --- | --- | --- | --- | --- | --- |
|  |  | N | % | N | % | N | % | N | % | N | % | N | % |
| **Dysphagia** | 16010 | 8690 | 54.28 | 3630 | 22.67 | 800 | 5.00 | 435 | 2.72 | 770 | 4.81 | 345 | 2.15 |
| **Diarrhea** | 1515 | 840 | 55.45 | 430 | 28.38 | 40 | 2.64 | 30 | 1.98 | 35 | 2.31 | 65 | 4.29 |
| **Constipation** | 9195 | 5015 | 54.54 | 2660 | 28.93 | 235 | 2.56 | 295 | 3.21 | 300 | 3.26 | 240 | 2.61 |
| **GERD** | 26930 | 14015 | 52.04 | 8160 | 30.30 | 790 | 2.93 | 645 | 2.40 | 845 | 3.14 | 610 | 2.27 |
| **Functional dyspepsia** | 135 | 65 | 48.15 | 50 | 37.04 | 0 | 0 | 0 | 0 | 5 | 3.70 | 0 | 0.00 |
| **IBS** | 1120 | 405 | 36.16 | 595 | 53.13 | 15 | 1.34 | 15 | 1.34 | 10 | 0.89 | 40 | 3.57 |
| **Incontinence** | 1100 | 55 | 5.00 | 255 | 23.18 | 70 | 6.36 | 55 | 5.00 | 45 | 4.09 | 15 | 1.36 |
| **UC** | 340 | 220 | 64.71 | 95 | 27.94 | 0 | 0.00 | 5 | 1.47 | 5 | 1.47 | 0 | 0.00 |
| **CD** | 370 | 210 | 56.76 | 120 | 32.43 | 0 | 0.00 | 15 | 4.05 | 0 | 0.00 | 0 | 0.00 |
| **Gastroparesis** | 575 | 185 | 32.17 | 290 | 50.43 | 25 | 4.35 | 25 | 4.35 | 5 | 0.87 | 20 | 3.48 |
| **Mortality** | 1645 | 885 | 53.80 | 415 | 25.23 | 60 | 3.65 | 45 | 2.74 | 55 | 3.34 | 25 | 1.52 |
| **NGT tube** | 590 | 330 | 55.93 | 135 | 22.88 | 35 | 5.93 | 10 | 1.69 | 35 | 5.93 | 10 | 1.69 |
| **PEG tube** | 2005 | 840 | 41.90 | 440 | 21.95 | 150 | 7.48 | 135 | 6.73 | 120 | 5.99 | 90 | 4.49 |
| **Malnutrition** | 5295 | 3310 | 62.51 | 645 | 12.18 | 450 | 8.50 | 485 | 9.16 | 370 | 6.99 | 885 | 16.71 |
| **Intestinal Obstruction** | 1005 | 585 | 58.21 | 165 | 16.42 | 70 | 6.97 | 25 | 2.49 | 50 | 4.98 | 20 | 1.99 |

Figure .S1 Prevalence of GI symptoms in patients with Parkinson's disease over age groups.
